# Cell-penetrating peptide functionalized ZIF-90 platform for photodynamic-chemical combined therapy in colorectal cancer

**DOI:** 10.1080/10717544.2026.2746812

**Published:** 2026-11-07

**Authors:** Tingting Fu, Boran Ni, Dongge Yin, Rongyue Zhu, Shilang Liao, Yuji Du, Xiaohong Jing, Yufei Sun, Xixi Deng, Chenyao Wu, Xiwen Cao, Mengru Cai, Xingbin Yin

**Affiliations:** a Department of Endocrinology, Guang’ Anmen Hospital of China Academy of Chinese Medical Sciences, Beijing, China; b School of Chinese Materia Medica, Beijing University of Chinese Medicine, Beijing, China; c Institute of Basic Theory for Chinese Medicine, China Academy of Chinese Medical Sciences, Beijing, China

**Keywords:** ZIF-90, curcumin, colorectal cancer, cell-penetrating peptide, photodynamic therapy, chemotherapy

## Abstract

Colorectal cancer (CRC) is one of the major concerns of developed nations. The combination of different treatment modalities has been shown to be effective in improving tumor therapy efficiency and reducing side effects. However, the synergistic anti-tumor efficiency and highly responsive drug release still remain challenging. Curcumin (CCM) has a chemotherapeutic effect as well as significant photodynamic activity, which is a promising component for the treatment of tumors. Herein, we report a cell-penetrating peptide functionalized ZIF-90-based drug delivery platform (PEG-CPP2@NPs) for synergistic photodynamic-chemical combined therapy in colorectal cancer. Polyethylene glycol (PEG)-conjugated CPP2 coating obviously increased the uptake of nanoparticles in tumor cells and their accumulation in tumor tissues. We conducted *in vivo* and *in vitro* experiments to evaluate the anti-tumor effects of CCM-loaded multifunctional nanoparticles. The PEG-CPP2@NPs exhibited significant tumor inhibition. After laser irradiation treatment, the generation of reactive oxygen species (ROS), mitochondrial damage, and cell apoptosis, as well as the expressions of Bax/Bcl-2, cytochrome C, and caspase-3, were all significantly enhanced. The tumor suppression rates in mice across the treatment groups were 25.46% for CCM, 33.16% for NPs, 43.5% for CCM+L, 48.45% for NPs+L, 66.05% for PEG-CPP2@NPs, and 80.64% for PEG-CPP2@NPs+L. This indicates that the functionalized ZIF-90 platform, when combined with chemotherapy and photodynamic therapy, has a stronger anti-tumor effect. Overall, the novel drug-delivery platform designed herein has demonstrated significant potential in the treatment of colon cancer and has provided a new approach for enhancing anti-tumor efficacy.

## 1. Introduction

Global cancer data show that approximately one in six deaths is caused by cancer, of which colorectal cancer accounts for 9.7% of the total incidence and 9.4% of the total mortality, posing a serious threat to people's health (Sung et al. 2021). The occurrence of colorectal cancer is related to factors such as diet (Song et al. 2015), lifestyle (Lewandowska et al. 2022) and genetics (Sawicki et al. 2021), and the incidence of colorectal cancer in young people has gradually increased in recent years (Mauri et al. 2019). At present, traditional cancer treatments mainly include surgical resection, chemotherapy, and radiotherapy (Miller et al. 2022). Surgery is the main treatment for colorectal cancer, but one-third of patients are still facing the dilemma of deterioration and recurrence (Cubas et al. 2007). The therapeutic effect of chemotherapy and radiotherapy is constantly improving with the development of technology, but problems such as poor tumor targeting, easy drug resistance, and severe side effects are still existing challenges (Mishra et al. 2013). Innovative therapeutic means are needed to improve the efficiency of tumor treatment.

Phototherapy is a new type of tumor treatment, including photodynamic therapy (PDT) and photothermal therapy (PTT) (Li et al. 2020). PDT produces a large number of reactive oxygen species (ROS) to directly kill tumor cells through irradiating the photosensitizer with specific wavelengths of light and transmitting light energy to the surrounding ground-state oxygen molecules (Correia et al. 2021). PDT has the characteristics of accurate local irradiation, rapid onset of effect, repeatable treatment, and low side effects (Agostinis et al. 2011; Kwiatkowski et al. 2018), which can play a synergistic anti-tumor effect in combination with chemotherapy, immunotherapy, and other therapies (El-Hussein et al. 2020; Jiang et al. 2023). For example, the construction of a nanoparticle drug delivery system to realize the synergistic treatment of Gambogic acid-chemotherapy and Ce6-PDT is an effective strategy to improve the activity of drugs and enhance the anti-tumor effect (Lan et al. 2021).

Photosensitizers are essential for PDT, and they are mainly divided into porphyrins and porphyrin derivatives and non-porphyrins in structure, among which curcumin is a natural source photosensitizer with excellent photodynamic properties (Dabrowski and Arnaut 2015; Correia et al. 2021). In addition, Curcumin (CCM) is a natural polyphenol active component mainly derived from *Curcuma longa* L. (Fuloria et al. 2022), which has good anticancer activity against colorectal cancer (Weng and Goel 2022; Idoudi et al. 2023), liver cancer (Dai et al. 2013; Marquardt et al. 2015), lung cancer (Wu et al. 2016; Ashrafizadeh et al. 2020), and breast cancer (Banik et al. 2017; Giordano and Tommonaro 2019). It has been reported that CCM can promote tumor apoptosis through generating ROS and regulating various signaling pathways, such as Bcl-2/Bax, Cyt C, and caspase 3 (Ismail et al. 2019; Wang et al. 2023). CCM has not only the anti-tumor activity of chemotherapy drugs, but also the photodynamic properties of photosensitizers, which can play a synergistic anti-tumor effect, and stimulate the enthusiasm of scholars. Unfortunately, CCM has relatively miserable stability, poor solubility, fast metabolism in vivo, and low bioavailability, which limits its clinical application effect (Prasad et al. 2014; Khezri et al. 2021). The construction of multifunctional drug delivery systems using nanocarriers is an effective measure to improve drug bioavailability and enhance therapeutic effects (Wong et al. 2019; Raj et al. 2021).

Metal-organic frameworks (MOFs) are increasingly widely applied in the field of drug delivery research with the advantages of diverse composition, large specific surface area, easy surface functionalization, and good biocompatibility (Lawson et al. 2021; Yang et al. 2023). ZIF-90 is one of the representative materials of Zeolitic imidazolate frameworks (ZIFs), a subclass of MOFs, with zeolite-like topology and cage-like cavity that provides enough space for drug loading and improves drug stability (Yang et al. 2022; Zeng et al. 2023). In addition, ZIF-90 has simple synthesis conditions, good thermal stability, pH response, and easy surface modification, which have significant advantages in building a multi-functional drug delivery platform (Jiang et al. 2019; Chen et al. 2024), and is expected to be used as a carrier of CCM to improve the shortcomings of CCM itself. Targeted drug delivery based on nanocarriers is another attractive strategy in tumor therapy. Cell-penetrating Peptides (CPPs) are a type of small-molecule peptides with strong transmembrane transport abilities without breaking the cell membrane structure (Zhang et al. 2016; Khan et al. 2021). CPPs can selectively recognize tumor cells and carry drug preparations through the barrier of tumor cell membranes to increase the uptake of drugs by tumor cells, which facilitates the full play of anti-tumor effects of drugs (Qiu et al. 2019; Kong et al. 2022). The series of research results of our research group demonstrated that the specific targeting effect of CPP44 on HepG2 cells significantly enhanced the anti-liver cancer activity of polyphyllin I (Wang et al. 2023). The specific targeting effect of CPP33 on A549 cells significantly enhanced the combined anti-lung cancer efficacy of survivin siRNA and oridonin (Cai et al. 2023). The studies have shown that CPP2 (DSLKSYWYLQKFSWR) can specifically penetrate colon cancer Lovo cells, modify the surface of drug-loaded particles, and promote the internalization of NPs by Lovo cells through kinetoprotein-dependent and mesh protein-independent pathways, thereby achieving targeted drug delivery (Kondo et al. 2012; Wang et al. 2016). In addition, surface coatings such as polyethylene glycol (PEG) have important advantages in multifunctional nanobiomedicine research (Fam et al. 2020), which can provide conjugating sites for targeted ligands, while improving the dispersion of drug-carrying particles and enhancing the stability of circulation *in vivo* (Wang and Thanou 2010).

In order to allow full play to the combined anti-tumor effects of CCM chemotherapy and PDT, we constructed a multifunctional nanoparticle drug delivery system, as shown in Figure 1. ZIF-90 was used as the carrier material to prepare high-drug-loading nanoparticles, and then target-ligand PEG-CPP2 was modified on the surface of the nanoparticles. The prepared multi-functional nanoparticles PEG-CPP2@NPs have good photodynamic properties and tumor selectivity, and have shown excellent anti-tumor effects in vitro and in vivo pharmacodynamic studies. Although existing studies have shown that CCM possesses photodynamic anti-cancer properties, most evaluations remain restricted to in vitro investigations. While MOFs/ZIFs and CPPs have individually been investigated, the distinct advantage demonstrated experimentally by the present integrated platform lies in utilizing a single multifunctional natural molecule (CCM) simultaneously as both a potent chemotherapeutic agent and an active photosensitizer within a high-capacity carrier. Specifically, the cage-like framework of ZIF-90 endows the system with an ultrahigh CCM loading efficiency (~55.24 wt%) and acid-triggered drug release, while the surface PEG-CPP2 functionalization effectively surmounts the rapid systemic elimination and poor aqueous stability of CCM, conferring preferential internalization into colorectal tumor cells. This unifies dual chemo-photodynamic modalities into a single nanoplatform without the carrier complexity of multi-drug co-encapsulation. The mechanism by which CCM exerts the combined anti-tumor effect of chemotherapy and PDT at the cytotoxic level was explored from both cellular and animal aspects, providing a reference strategy for photosensitive and hydrophobic natural drugs. Regrettably, this study did not consider the possibility of curcumin exerting anti-cancer effects through its immunomodulatory potential, and failed to clarify the mechanism by which CCM exerts the combined anti-tumor effect of chemotherapy and PDT at the immunomodulatory level.

**Figure 1.**
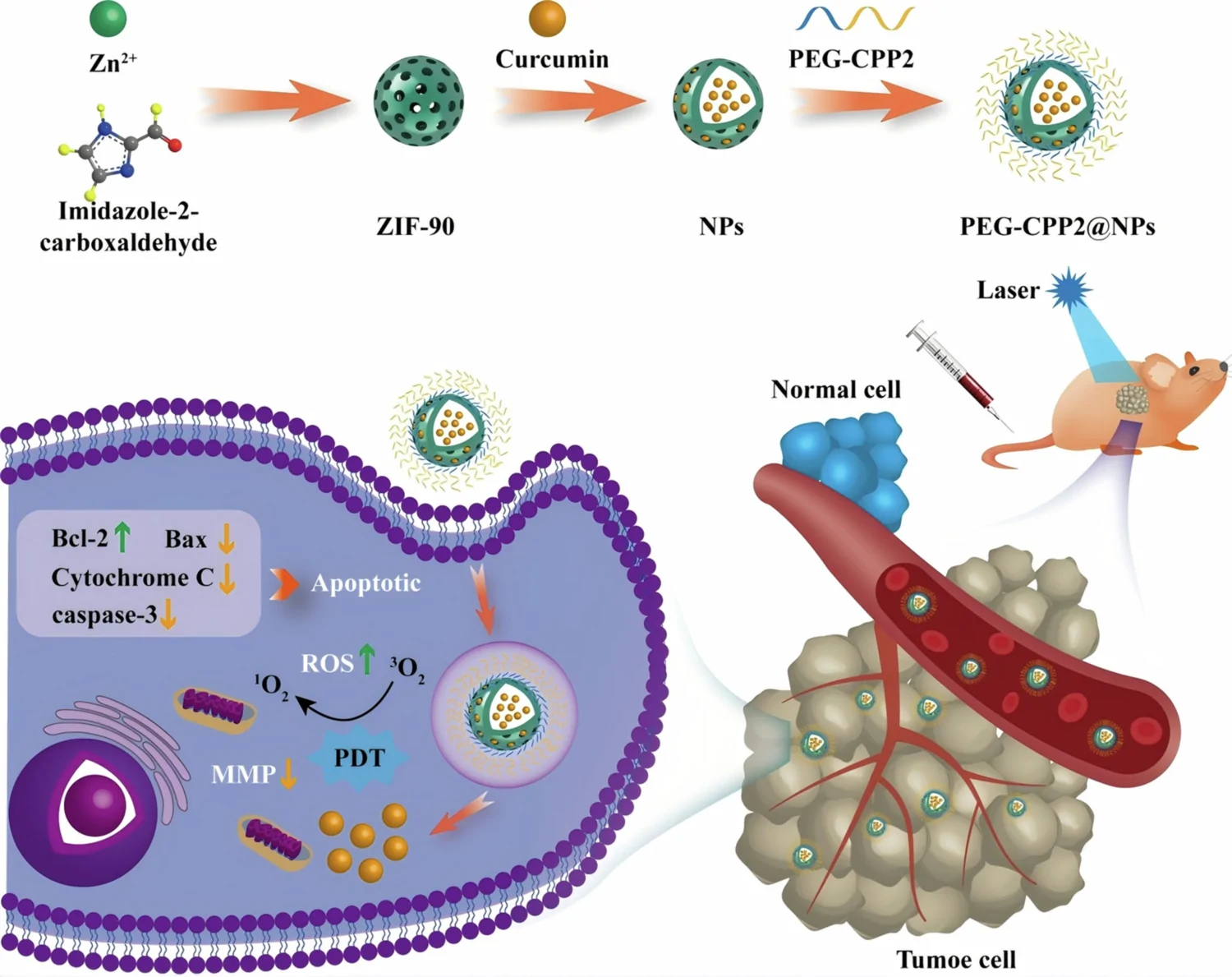
Schematic illustration of the construction of multifunctional metale-organic framework NPs and anti-tumor therapy.

## 2. Materials and methods

### 2.1. Materials

Curcumin (98%, Cat. No. S31628), Imidazole-2-formaldehyde (98%, Cat. No. I812136), 1,3-Diphenylisobenzofuran (DPBF, 97%, Cat. No. S48248) and Methylene Blue (MB, 98%, Cat. No. S19044) were obtained from Shanghai Yuanye Biotechnology Co., Ltd. Zn(CH_3_COO)_2_·2H_2_O (98%, Cat. No. Z820726) was supplied by Shanghai Macklin Biochemical Co., Ltd. *N*, *N*-dimethylformamide (DMF, 99.5%, CAS: 68-12-2) and Dimethyl sulfoxide (DMSO, CAS: 67-68-5) were provided by Tianjin Fuchen Chemical Reagent Factory. Methanol (CH_3_OH) was obtained from Thermo Fisher Scientific Technology Co., Ltd. RPMI-1640 medium (Cat. No. C11875500BT) and Phosphate Buffered Saline (PBS, Cat. No. C10010500BT) were purchased from Gibco. Fetal Bovine Serum (FBS, Cat. No. 11011-8611) were acquired from Beijing Sijiqing Biotechnology Co., Ltd. 3-(4,5-Dimethylthiazol-2-yl)-2,5-dipheny-ltetrazoliumbromide (MTT, Cat. No. ST316), DAPI dye (Cat. No. C1006), Annexin V-FITC Apoptosis Detection Kit (Cat. No. C1062L), Reactive oxygen species detection kit (Cat. No. S0033S), Mitochondrial membrane potential detection kit (JC-1) (Cat. No. C2006) and Cell Cycle and Apoptosis Analysis Kit (Cat. No. C1052) were purchased from Shanghai Beyotime Biotechnology Co., Ltd. Tunel Cell Apoptosis Detection Kit (Cat. No. G1504) was supplied by Wuhan Servicebio Technology Co., Ltd. Tissue ROS test kit (DHE, Cat. No. HR8821) was obtained from Beijing Baiaolaibo Technology Co., Ltd. Anti-GAPDH antibody (Cat. No. ab9485), Anti-Bax antibody (Cat. No. ab32503), Anti-Bcl-2 antibody (Cat. No. ab182858), Anti-Cytochrome C antibody (Cat. No. ab133504) and Anti-Caspase-3 antibody (Cat. No. ab184787) were purchased from Abcam (Shanghai, China). ColorMixed protein marker (11-180 KD) (Cat. No. BN27001) was obtained from Biorigin (Beijing) Inc.

### 2.2. Preparation of ZIF-90

Imidazole-2-formaldehyde (0.4 M) and Zn(CH_3_COO)_2_·2H_2_O (0.4 M) were completely dissolved in DMF, respectively. Mixed 2 mL of Imidazole-2-formaldehyde solution with 1  mL of Zn(CH_3_COO)_2_·2H_2_O solution and stirred for 10 min, and then 10  mL DMF was added and stirred for 30 min. The products were collected by centrifugation for 30 min, and the obtained precipitations were washed twice with methanol and dried in a vacuum drying oven at 60 °C for 24 h. The above-mentioned experimental study period was from February 10, 2023 to March 8, 2023.

### 2.3. CCM loading

The prepared ZIF-90 was added to the CCM solutions and magnetically stirred in the dark. The drug-loading nanoparticles (CCM@ZIF-90), denoted as NPs, were collected by centrifugation, and drug loading was determined by high-performance liquid chromatography (HPLC). In addition, we selected three factors that affect the drug loading, namely, the drug loading time (12, 24, and 48  h), the drug loading ratio (1:1, 2:1, and 4:1), and the drug loading concentration (2, 3, and 4 mg/mL), and employed L_9_ (3^4^) orthogonal design to determine the optimal drug-loading process. The above-mentioned experimental study periods were from February 10, 2023 to May 24, 2023.

### 2.4. Preparation of PEG-CPP2@NPs

NPs and PEG-CPP2 were evenly mixed in purified water at a mass ratio of 5:1, and stirred at 600  rpm for 30  min in the dark. The products were centrifuged for 30 min, lyophilized for 24 h, and stored at -20 °C. The above-mentioned experimental study period was from May 16, 2023 to May 28, 2023.

### 2.5. Particle characterization

The morphologies and structures of the prepared NPs were observed using scanning electron microscopy (SEM, Hitachi S-4700, Japan). The particle size distribution of NPs was surveyed using a dynamic light scattering analyzer (DLS, Malvern Nano Zetasizer S90, Malvern, UK). The structure of the characteristic functional groups was analyzed by a Fourier-transform infrared spectrometer (FTIR, Thermo Fisher Nicoler-6700, USA). The crystal structure was identified by X-Ray diffractometer (XRD, Rigaku Ultima IV, Japan). The BET surface area, pore volume, and pore diameter of particles were determined by Brunauere–Emmette–Teller sorptometer (BET, Micromeritics ASAP 2460, America). The thermal stability of the materials was investigated by a thermogravimetric analyzer (TGA, Shimadzu DTG-60AH, Japan). The above-mentioned experimental study periods were from February 22, 2023 to June 24, 2023.

### 2.6. *In vitro* drug release investigation

The release behaviors of CCM, NPs, and PEG-CPP2@NPs in vitro at pH 7.4 and 5.5 were investigated by the centrifugal method. The samples were oscillated at 37 °C and 100  rpm for 24 h. 1 mL of the sample was centrifuged at 0, 15 and 30 min, as well as at 1, 2, 4, 6, 8, 10, 12 and 24 h, and the supernatant was collected. Then, 1 mL of fresh culture medium was added. The CCM content in the collected samples was detected by HPLC, the cumulative release rate was calculated and the release curve *in vitro* was drawn. The above mentioned experimental study periods were from May 29, 2023 to June 15, 2023.

### 2.7. Photostability and photodynamic properties investigation

Under dark conditions, CCM, NPs and PEG-CPP2@NPs solution (10 μM CCM) were irradiated with a 450 nm fiber-coupled laser at 7.6  mW/cm^2^ for 60 min. The absorbance at 430 nm at different time points (0, 1, 2, 3, 4, 5, 10, 15, 20, 25, 30, 40, 50, and 60 min) was measured by a ultraviolet spectrophotometer to evaluate the photostability. DMF was used to configure the DPBF solution (3.6 × 10^−5^ mM), MB solution (5 × 10^−6^ mM), and CCM nanomaterials solution (5 × 10^−6^ mM CCM). Next, 10  μL of MB and CCM nanomaterials solution were added to 3  mL DPBF solution, respectively, and laser irradiated for 3 min. The absorbance at 415 nm was recorded by a ultraviolet spectrophotometer every 10 s to calculate singlet oxygen yield. In a black 96-well plate, 150  μL of 0.04  mM DCFH solution was first added, and then 150  μL of CCM nanomaterials solution (10  μM CCM) was added, respectively. The fluorescence intensity of each sample was detected by a multifunctional enzyme marker before and after 3 min of laser irradiation for each well to evaluate the ROS yield. The excitation wavelength of the detector was 488 nm, and the emission wavelength was 525 nm. The above-mentioned experimental study periods were from June 27, 2023 to January 16, 2024.

### 2.8. *In vitro* cellular uptake

A Lovo cell line was purchased from BeNa Culture Collection, Henan, China, and HepG2 cell line was purchased from Guangzhou Jenniobio Biotechnology Co., Ltd. Guangzhou, China. MCF-7 cells and HL-7702 cells were obtained from the China Infrastructure of Cell Line Resources. The cells were cultured in RPMI-1640 medium containing 10% (v/v) fetal bovine serum and 1% (v/o) penicillin-streptomycin and incubated in an incubator at 37 °C with 5% CO_2_.

The cellular uptake of CCM-NPs by Lovo, HepG2, MCF-7, and HL-7702 cells was analyzed using Confocal Laser Scanning Microscope (CLSM). The cells were inoculated in petri dishes for 36  h and incubated with 1 mL CCM-NPs solution at CCM concentrations of 15  μg/mL for 4  h. Then, cells were washed three times with 1 mL PBS, fixed with 0.5  mL 4% paraformaldehyde for 10  min, and imaged with CLSM. Subsequently, the fluorescent images of the cells were quantitatively analyzed using ImageJ. To further analyze the cellular uptake of PEG-CPP2@NPs in Lovo cells, the cells were incubated with CCM-NPs (CCM: 15  μg/mL) for 4 h. Lovo cells were then collected and rinsed, and the CCM fluorescence intensity was measured by flow cytometry. The above-mentioned experimental study periods were from June 1 st, 2023 to June 27th, 2023.

### 2.9. *In vitro* cytotoxicity assay

Lovo cells were inoculated in 96-well plates at a density of 8000 cells per well and cultured for 36 h before adding 100  μL medium containing different concentrations of CCM-NPs (CCM: 5, 10, 15, 20, and 25 μg/mL). After the cells were cultured for 8 h, the fresh medium was replaced and treated with or without 450 nm laser irradiation (71.3  mW/cm^2^) for 3 min. Then, the cells were continuously cultured for 16 h. MTT (100  μL, 1 mg/mL) solution was added, and the mixture was further incubated for 4 h. The medium was discarded, and 100  μL DMSO was added to each well. The samples were shaken for 5 min under conditions of avoiding light, and the absorbance at 490 nm was measured with an Enzymograph. The above-mentioned experimental study periods were from March 18, 2023 to June 10, 2023.

### 2.10. Cell apoptosis assay

The degree of cell apoptosis induced by different NPs was detected by flow cytometry. Lovo cells were inoculated in 6-well plates at a density of 3 × 10^5^ cells per well for 36 h and treated with 1 mL CCM-NPs solution (CCM: 20  μg/mL) for 8 h. Then, the cells were replaced with fresh medium, and the laser irradiation groups were irradiated for 3 min. After continued incubation for 16 h, the cells were collected and resuspended in 295 μL binding buffer. Then, the cells were stained with 5  μL Annexin V-FITC for 15 min and 10 μL PI for 5  min, and the fluorescence intensity was analyzed using flow cytometry (FACSCanto II, USA). The above-mentioned experimental study periods were from June 1, 2023 to June 21, 2023.

### 2.11. Reactive oxygen species (ROS) detection

Lovo cells were treated with CCM-NPs at CCM concentrations of 20  μg/mL for 8 h. The treated cells were washed with PBS and irradiated with or without a laser for 3 min. After incubation with 1 mL DCFH-DA (10 μmol/L) for 30  min, imaging was performed under a fluorescence microscope. In addition, ROS quantitative analysis was performed by flow cytometry. The above-mentioned experimental research periods were from June 1, 2023 to July 19, 2023.

### 2.12. Detection of mitochondrial membrane potential (MMP)

The changes in MMP were measured using JC-1 fluorescent probes to evaluate mitochondrial damage. Lovo cells were treated with CCM-NPs at CCM concentrations of 20  μg/mL for 8 h. Subsequently, the cells were replaced with fresh medium, and the cells in the laser irradiation groups were irradiated for 3 min. After continued incubation for 16 h, the cells were collected and resuspended in 0.5 mL cell culture medium. Then, the cells were stained with 0.2  mL JC-1 for 30  min, rinsed, and analyzed by flow cytometry. The above-mentioned experimental study periods were from July 8, 2023, to July 23, 2023.

### 2.13. Cell cycle detection

The effect of CCM-NPs on the Lovo cell cycle was analyzed by Propidium (PI) staining. Lovo cells were treated with ZIF-90 and CCM-NPs at CCM concentrations of 20  μg/mL for 8  h. After replacing the fresh medium, the culture continued for 16 h. The cells were incubated with 0.5  mL PI for 30  min, washed, and detected by flow cytometry. The above-mentioned experimental study periods were from August 2, 2023 to August 18, 2023.

### 2.14. Animal and tumor-bearing mouse model

All the animal experiments were conducted in accordance with the guidelines approved by the Institutional Animal Care and Use Committee of the Beijing University of Chinese Medicine (BUCM-2023101802-4068). BALB/c nude mice (5–6 weeks, 20 g) were acquired from Sipeifu Biological Technology Co. Ltd. (Beijing, China) for establishing tumor-bearing mouse models. Lovo cells suspension (2 × 10^6^ cells) was subcutaneously injected into the right underarm of the mice, and the tumor volume (V = W^2^ × L/2) was monitored daily. Approximately 2 weeks after Lovo cells inoculation, the tumor volume in the BALB/c nude mice approached 150 mm^3^.

In adherence to the 3Rs principles (Replacement, Reduction, and Refinement) of animal ethics, the tumor-bearing mice were divided into two groups for the biodistribution study (*n* = 3 per group) and seven groups for the *in vivo* anti-tumor efficacy study (*n* = 4 per group). Although non-parametric statistical methods were employed, we acknowledge that an animal sample size of *n* = 4 offers limited statistical power, which represents a recognized constraint of the present *in vivo* study when drawing generalized conclusions regarding therapeutic efficacy and safety. When studying the biological distribution of nanomedicines, tumor-bearing mice were divided into two groups: the nanoparticle group (Cy5.5@NPs) and the targeted nanoparticle group (PEG-CPP2@Cy5.5@NPs), with 3 mice in each group, to investigate the tissue distribution of nanoparticles in the body. When studying the *in vivo* anti-tumor effect of nanomedicines, tumor-bearing mice were divided into 7 groups: saline control group, non-light groups (CCM, NPs, PEG-CPP2@NPs), and light groups (CCM+L, NPs+L, PEG-CPP2@NPs+L), with 4 mice in each group, to confirm the anti-tumor efficacy of the nanomedicines.

At the end of the experiment, a total of 34 mice used during the experiment were euthanized. All the mice were euthanized at the end of the experiment. First, the mice were subjected to deep anesthesia with isoflurane (confirmed by the disappearance of the toe clip reflex), and then, the cervical dislocation was rapidly treated by trained personnel. After confirming the death of the mice through dilated pupils and cessation of breathing and heartbeat, blood, organ and tumor collections were carried out.

### 2.15. *In vivo* fluorescence imaging and bio-distribution analysis

*In vivo* biological distribution and tumor accumulation of fluorescent-labeled NPs (Cy5.5@NPs, PEG-CPP2@Cy5.5@NPs) were investigated using the IVIS imaging system. NPs were injected intravenously into tumor-bearing mice, and the mice were imaged *in vivo* at 0, 2, 4, 6, 12, and 24  h after injection. Twenty-four hours after administration, the mice were anesthetized and euthanized. Samples of the heart, liver, spleen, lungs, kidneys, and tumors were collected for *in vitro* tissue imaging. The fluorescence intensity was quantitatively analyzed using Living Image software. The above-mentioned experimental study periods were from September 21, 2023 to December 15, 2023.

### 2.16. *In vivo* anti-tumor effect and biosafety analysis

BALB/c nude mice with tumors of almost 150 mm^3^ were randomly divided into 7 groups (random grouping was performed using SPSS 22.0 software): saline control group, non-light groups (CCM, NPs, PEG-CPP2@NPs), and light groups (CCM+L, NPs+L, PEG-CPP2@NPs+L). On the 1st, 3rd, 5th, and 7th days after randomization, intravenous injection was administered to each group, and all preparations were administered at a CCM dose of 7.5  mg/kg. In the light group, the mice were treated with laser irradiation (0.5  W/cm^2^) for 5  min at 6  h after injection. The weight and tumor volume of the mice were measured daily during treatment. On the 8th day of treatment, blood, tumors, and major organs (heart, liver, spleen, lungs, and kidneys) were collected from the mice. Half of the tumor tissue was used for the ROS assay and Western blot (WB) detection, and the other half was preserved in 4% formaldehyde for hematoxylin and eosin (HE) staining and terminal deoxynucleotidyltransferase-mediated nick end labeling (TUNEL) staining. The above-mentioned experimental study periods were from September 21, 2023 to January 25, 2025.

### 2.17. *In vivo* biosafety analysis

To analyze the biological safety of nanoparticles in the body, the serum biochemical indicators in the blood of mice were detected, including AST, ALT, ALP and BUN. Furthermore, the major organs of the heart, liver, spleen, lung, and kidney were stained with HE. The above-mentioned experimental study periods were from December 12, 2023 to January 25, 2025.

### 2.18. Statistical methods

All experiments were performed with at least three independent samples, and all results were expressed as means ± SD. Data analysis was performed through GraphPad Prism 9.0 (GraphPad Software, USA) using Student's t-test or one-way ANOVA. Statistical significance was considered as *P < 0.05, **P < 0.01, and ***P < 0.001.

## 3. Results and discussion

### 3.1. Preparation of ZIF-90

In DMF reaction medium, imidazole-2-formaldehyde as an organic ligand and metallic Zn were self-assembled to form Zeolitic imidazolate frameworks (ZIF-90) (Li et al. 2022). As illustrated in Figure 2A, SEM was used to analyze the structure and morphology of the prepared ZIF-90. The ZIF-90 was clearly quasi-circular and smooth, with a particle size of approximately 60 nm. The average specific particle size measured by DLS was 63.58 ± 2.74 nm (Fig. S1), which was consistent with the SEM results.

As shown in Figure 2B, the FTIR spectra of the ZIF-90 showed characteristic peaks at 2930 cm^−1^ and 2840 cm^−1^ (C─H stretching of aldehyde group) and 1678 cm^−1^ (C═O stretching of aldehyde group), confirming the presence of aldehyde group in the organic ligand imidazole-2-formaldehyde. The peaks at 1400–1200 cm^−1^ became sharp, indicating that the coordination environment belonged to *N* coordination, and it was speculated that the *N* coordination site of imidazole-2-formaldehyde had undergone a coordination reaction with Zn^2+^. The peak at 800–600 cm^−1^ was the out-of-plane C─H bending vibration peak of the imidazole molecular ring. The XRD pattern showed that ZIF-90 had an obvious peak pattern and good crystallinity, with typical diffraction peaks at 6.7°, 9.7°, 12.0°, 14.0°, 15.8°, and 17.3° (Figure 2C). The results of TGA showed that ZIF-90 could maintain the relative stability of the skeleton in the range of 300 °C, and had good thermal stability (Figure 2D). The mass loss of ZIF-90 at 0–200 °Cwas approximately 10 wt%, which may be caused by the evaporation of the adsorbed water on the surface of ZIF-90 and the residual reaction medium DMF in the internal channel. After 300 °C, there was a continuous, obvious weight loss, which was caused by the gradual pyrolysis of the bonds in the organic molecular chains caused by high temperature, causing the collapse of the skeleton.

In addition, BET was used to determine the pore structure parameters and pore size distribution of ZIF-90. BET and BJH curves showed that the BET surface area of ZIF-90 was 1131.89 m^2^/g, the pore volume was 1.24  cm^3^/g, and the pore diameter was 4.3987 nm (Figure 2E and F). These results indicate that ZIF-90, a MOF with a small particle size, clear structure, complete crystal form, and good thermal stability, has been successfully synthesized. At the same time, it has a large specific surface area, sufficient pore volume, and uniform distribution, which was suitable as a carrier material for drug delivery.

**Figure 2.**
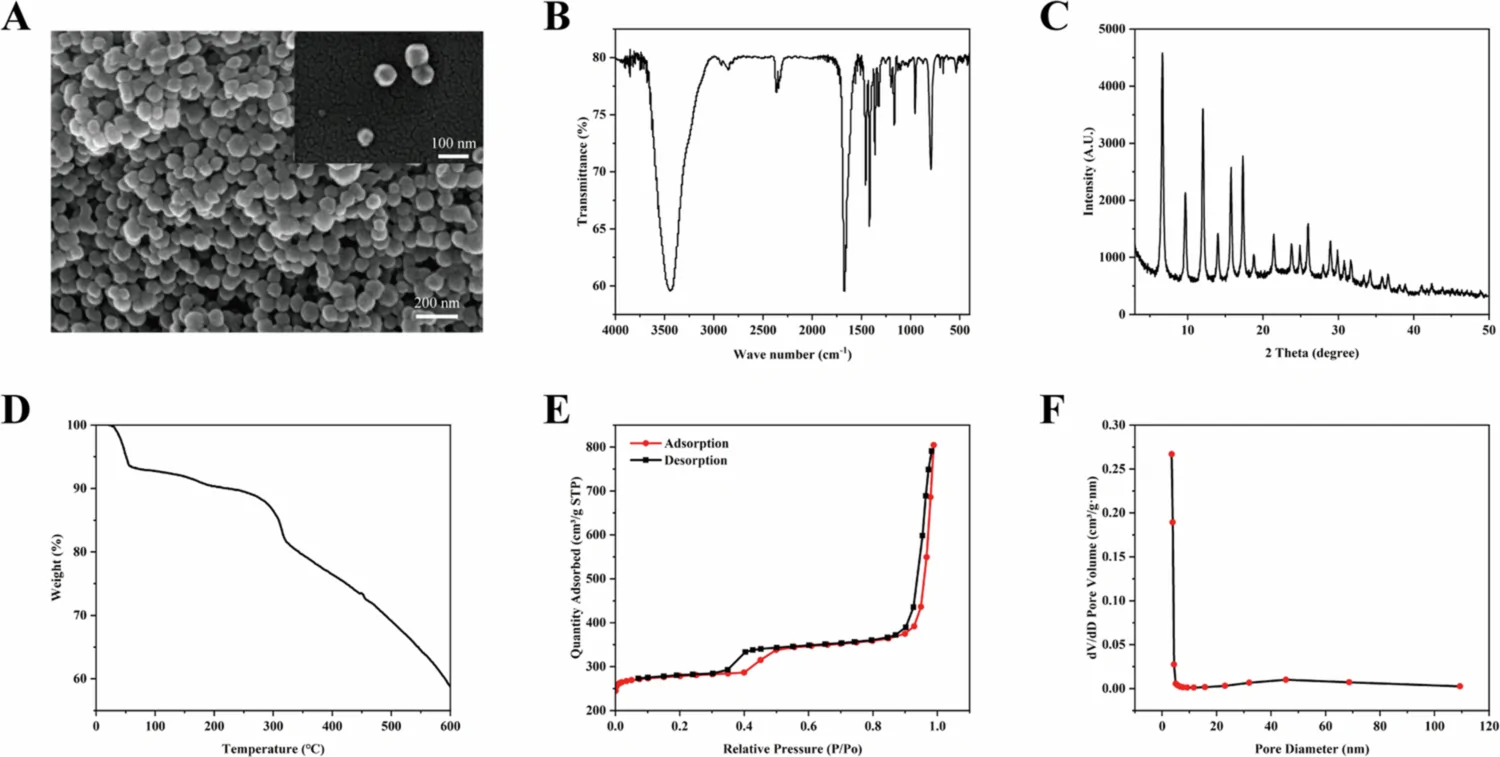
Characterization of the structure and properties of ZIF-90. (A) SEM, (B) FTIR, (C) XRD, (D) TGA, (E) BET isotherm, (F) aperture distribution curve.

### 3.2. Drug loading

Ex situ encapsulation was used as a method for loading CCM with ZIF-90. Based on the references (Sun et al. 2011) and the previous research foundation (Cai et al. 2022), taking the drug loading quantity as the evaluation index and the drug loading time, drug-loading ratio and drug concentration as the influencing factors, the L_9_(3^4^) orthogonal design was used to optimize the drug-loading process and explore the optimal proportion of CCM loaded with ZIF-90 (Tables S1, S2, and S3). The results showed that when the drug-loading time was 48 h, the drug-to-carrier ratio was 4:1, and the CCM concentration was 3 mg/mL, the drug-loading capacity was the highest. Three batches of verification experiments were carried out (Table S4). The drug-loading capacity of CCM@ZIF-90 NPs was 55.24 ± 1.94  wt%, which proved that the drug-loading process was stable and feasible. The high loading capacity of ZIF-90 on CCM was attributed to its large specific surface area and pore volume.

According to the SEM results, when the loaded CCM of ZIF-90 becomes CCM@ZIF-90 NPs, its shape did not change, but its particle size was slightly larger than that of ZIF-90 (Figure 3A). The mean particle size of the CCM@ZIF-90 NPs measured by DLS was 82.34 ± 5.72  nm (Figure S2). As shown in Figure 3B, the characteristic peaks of the FTIR spectra of ZIF-90 blank material were roughly similar to those of CCM@ZIF-90 NPs, indicating that the skeleton structure of the material was not damaged during drug loading. Curcumin is a polyphenol compound with a *β*-dione structure, and its FTIR spectra showed the following characteristic peaks: 1630  cm^−1^ (C═O stretching of the unsaturated carbonyl group), 1603, 1510, and 1427  cm^−1^ (C═C stretching of the benzene ring), 1280 and 1028  cm^−1^ (C─O stretching of aromatic ether), and 1118  cm^−1^ (C─C skeleton vibration of C─C═O). The FTIR spectra of CCM@ZIF-90 NPs showed multiple vibration peaks from curcumin at 1510, 1280, 1118, and 1028  cm^−1^, indicating the existence of CCM in the drug-loaded NPs. The XRD results showed that the characteristic diffraction peaks of CCM@ZIF-90 NPs were at 7.0°, 10.0°, 12.4°, 14.5°, 16.1°, and 17.7° (Figure 3C). The diffraction peak of the drug-loaded NPs was similar to ZIF-90, but the overall peak position was shifted 0.3°–0.5° to a higher angle, which meant that the lattice constant became smaller, and heteroatoms with a smaller radius than the main atom might be incorporated. According to BET results (Figure 3D and E), the specific surface area and pore volume of the CCM@ZIF-90 NPs were 238.57 m^2^/g and 0.32  cm^3^/g, respectively, both of which were significantly smaller than those of the blank material ZIF-90. The above characterization results showed that CCM was encapsulated in ZIF-90 by pore encapsulation and surface adsorption.

In addition, TGA was performed on CCM and drug-loaded NPs. As shown in Figure 3F, the weight of CCM rapidly decreases from 250 °C, which may be due to the instability of CCM at high temperature and chemical decomposition. At 250–300 °C, the weight loss of CCM@ZIF-90 NPs was 5.54 wt%, which was higher than that of ZIF-90 by 3.04 wt%, which may be caused by the decomposition of drugs adsorbed by ZIF-90 in a shallow layer, indicating that CCM had been loaded in ZIF-90. At 300–400 °C, the weight loss of CCM@ZIF-90 NPs was 11.58 wt %, while the weight loss of CCM was as high as 35.65  wt%, indicating that the inclusion of ZIF-90 had a protective effect on CCM, and that the drug-loaded NPs had better thermal stability than the CCM. In conclusion, the above characterization results illustrated that CCM@ZIF-90 NPs had been successfully prepared.

**Figure 3.**
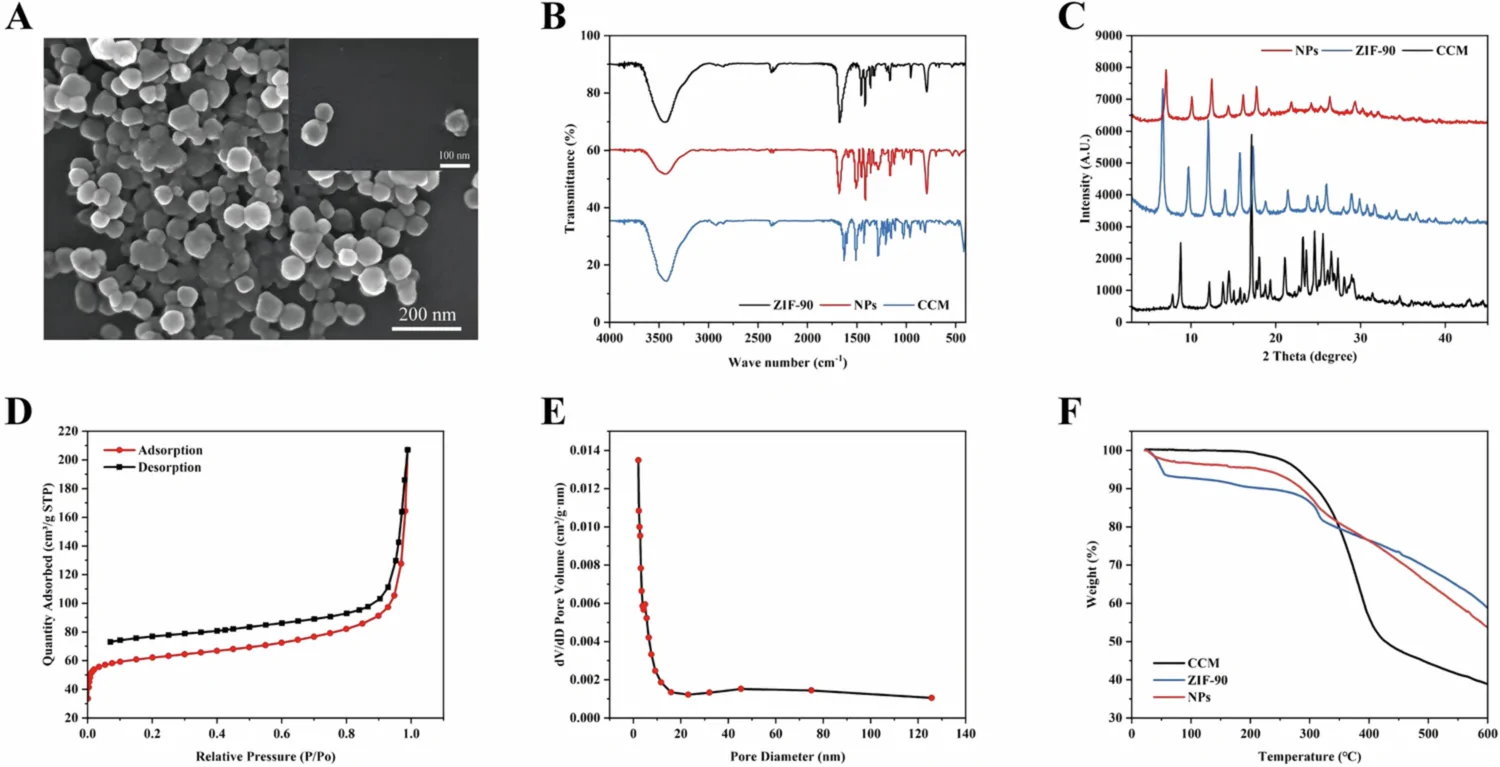
Characterization of the structure and properties of CCM@ZIF-90. (A) SEM, (B) FTIR, (C) XRD, (D) BET isotherm, (E) aperture distribution curve, (F) TGA.

### 3.3. Preparation and characterization of PEG-CPP2@NPs

To achieve targeted drug delivery and improve delivery efficiency, we selected CPP2, which can specifically penetrate colon cancer cells, as a targeted peptide, and formed a stable thioether bond through the addition reaction of a sulfhydryl group at the end of CPP2 and a maleimide group at the end of mPEG2000-Mal to construct the targeting ligand PEG-CPP2. According to the method of stirring at room temperature, by virtue of the hydrogen bond coating between PEG and ZIF-90, the PEG-CPP2 was modified on the surface of CCM@ZIF-90, and the targeted drug-loaded NPs (PEG-CPP2@NPs) were prepared, so as to improve the dispersion of the NPs, stability of internal circulation, and aggregation in tumor cells.

As shown in Figure 4A, after modifying the targeted ligand with drug-loaded NPs, it can be seen that substances were coated on the surface of the NPs, and the particle size was further increased. The specific particle size was 109.13 ± 2.48 nm (Fig. S3). The Zeta potential of PEG-CPP2@NPs was approximately -29 mW, which was as negative as that of CCM@ZIF-90, but the potential value of targeted drug-loaded NPs was much higher than that of the drug-loaded NPs (Figure S4), indicating that PEGylation increased the surface charge of NPs and improved its dispersion. In the FTIR spectrum (Figure 4B), some characteristic peaks of PEG-CPP2 appeared in the targeted drug-loaded NPs at 2860 and 845  cm^−1^. The BET results showed that the specific surface area and pore volume of PEG-CPP2@NPs were 39.00 m^2^/g and 0.25  cm^3^/g, respectively, both of which were significantly smaller than CCM@ZIF-90 (Figure 4C and D). These results illustrated that PEG-CPP2 had been successfully modified on the surface of CCM@ZIF-90. In addition, the XRD pattern of PEG-CPP2@NPs was further examined, and it was found that the characteristic peak of NPs did not change significantly after PEGylation, only the peak strength was slightly weakened (Figure 4E), indicating that PEGylation had no effect on the crystal form of NPs.

Drug release is an important research focus. The release behavior of free drugs and NPs in acidic (pH = 5.5) and neutral (pH = 7.4) environments was investigated by centrifugal method. As shown in Figure 4F, the cumulative release rate of free CCM in the two pH environments exceeded 60% within 1 h, while the drug release characteristics of PEG-CPP2@NPs and CCM@ZIF-90 NPs were similar, and the cumulative release rate reached the maximum in about 12 h. At pH 5.5, the cumulative release rates of CCM in PEG-CPP2@NPs and CCM@ZIF-90 within 24 h were 65.28 ± 3.64% and 61.05 ± 3.48%, respectively. In contrast, the cumulative release rates of CCM in the two NPs were 54.47 ± 1.96% and 51.71 ± 6.80%, respectively, at pH 7.4. The CCM cumulative release rate of the two NPs at pH 5.5 was approximately 10% higher than that at pH 7.4, showing a statistical difference. Compared with free CCM, the two NPs showed certain slow-release properties and pH responsiveness.

**Figure 4.**
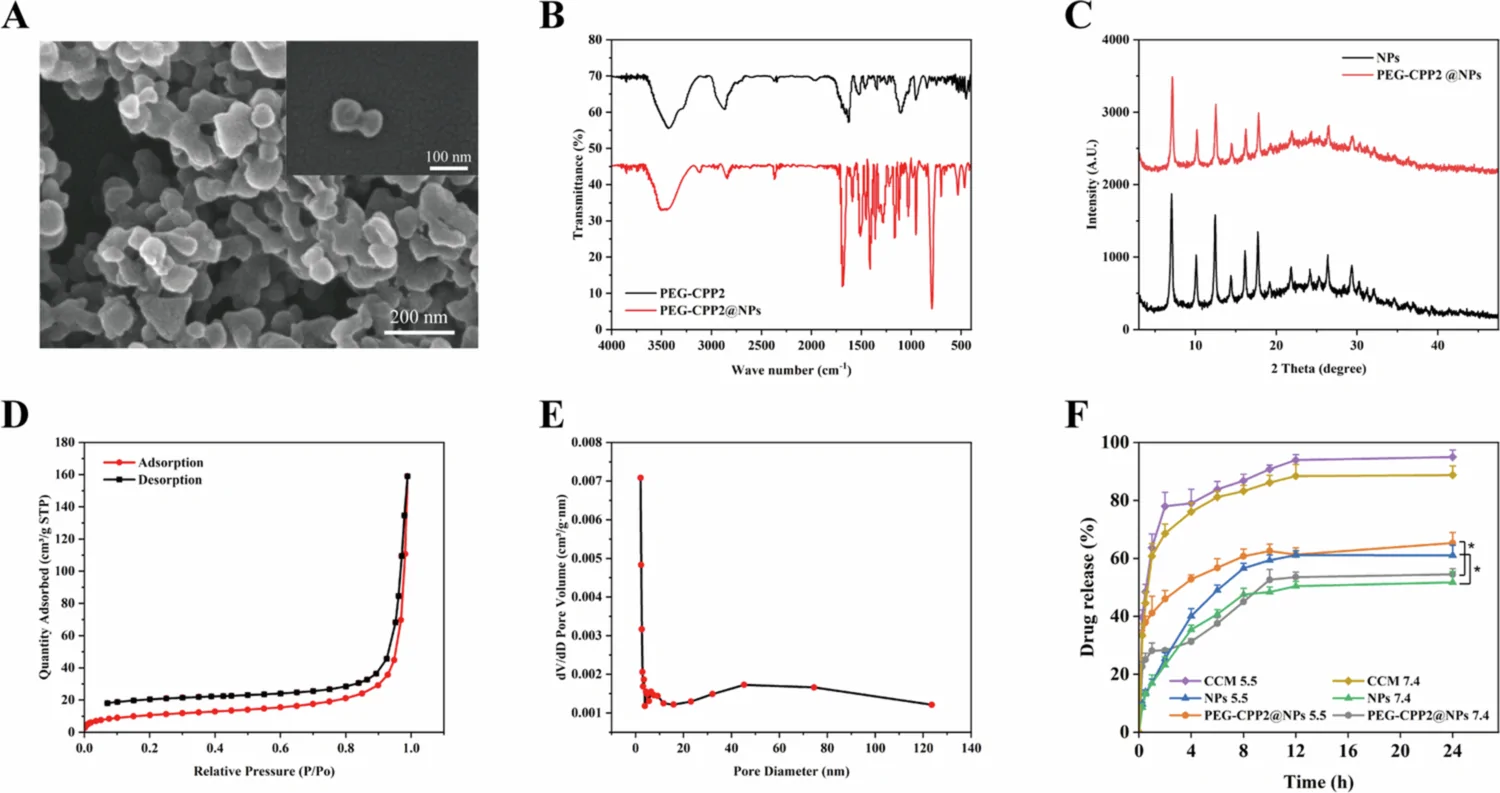
(A) SEM, (B) FTIR, (C) XRD, (D) BET isotherm, and (E) aperture distribution curve of PEG-CPP2@NPs, (F) release of free CCM, CCM@ZIF-90, and PEG-CPP2@NPs (*n* = 3, *P < 0.05, ***P < 0.001).

### 3.4. Investigation of photostability and photodynamic properties

The photodynamic properties of photosensitive substances are the basis of photodynamic therapy, and the photostability may affect the efficiency of PDT. The optical stability of CCM and its nano-preparations was evaluated using UV spectrophotometry to measure the absorbance of CCM at 430 nm after different times of laser irradiation (Figure 5A). The molar extinction coefficient of curcumin under 450 nm light irradiation was 5.52 × 10^4^ M^−1^·cm^−1^. In the absence of laser irradiation, the absorbance of free CCM remained almost unchanged, while the free CCM degraded by 33.39% after 60 min of laser irradiation. Under the condition with laser, the absorbance of CCM@ZIF-90 and PEG-CPP2@NPs has the same time-dependent decrease as free CCM, but the degradation rates were 19.82% and 17.83%, respectively. This indicated that the nano-preparations had stronger photostability than free CCM.

After the photosensitizer was irradiated by a specific wavelength of light, the light energy can be transferred to the surrounding oxygen molecules, resulting in reactive oxygen species such as singlet oxygen (^1^O_2_), hydrogen peroxide (H_2_O_2_), superoxide anion (·O^2-^), hydroxyl radical (·OH), etc. 1,3-Diphenylisobenzofuran (DPBF) has a characteristic absorption peak at 415 nm because of its conjugated structure, and the ring-opening reaction occurs in the presence of singlet oxygen, which decreases the absorbance of the characteristic absorption peak. We used DPBF as ^1^O_2_ trapping agent and methylene blue (MB) as a positive reference substance to detect singlet oxygen yield of CCM and NPs by chemical spectrophotometry. The results were shown in Figure 5B, the photocatalytic oxidation rates of DPBF by CCM and NPs were higher than MB, and the singlet oxygen yield of MB in DPBF was 49.1%, while the singlet oxygen yields of CCM, CCM@ZIF-90, and PEG-CPP2@NPs were 65.1%, 62.6%, and 63.1%, respectively, suggesting that CCM and its NPs had good photodynamic activity *in vitro*. In addition, using DCFH as a fluorescent probe, the fluorescence intensity was detected by a multifunctional enzyme marker to evaluate ROS production. As shown in Figure 5C, the ROS production of CCM and its NPs after laser irradiation was obviously higher than that without laser irradiation, exhibiting good photodynamic performance.

**Figure 5.**
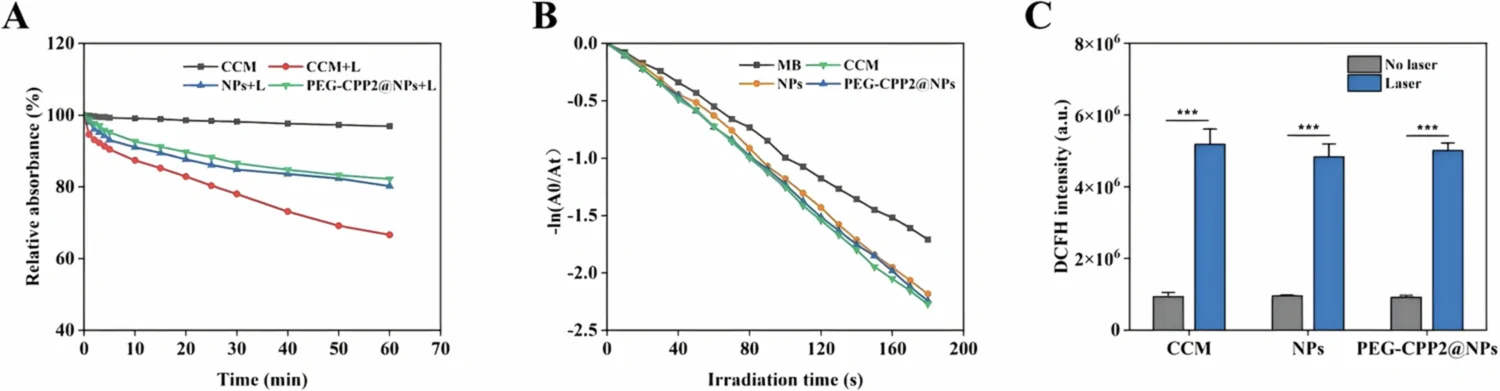
(A) Photostability, (B) Singlet oxygen yields and (C) ROS production of CCM, NPs and PEG-CPP2@NPs (*n* = 3, *P < 0.05, ***P < 0.001).

### 3.5. Cellular uptake

The specific selection of drugs for tumor cells is the key to targeted therapy. Confocal laser scanning microscopy (CLSM) was used to study the targeting selectivity of PEG-CPP2-modified NPs to different types of cells. The results showed that all four kinds of cells appeared green fluorescence after incubation with CCM and CCM@ZIF-90, indicating that the selectivity of CCM and CCM@ZIF-90 was not obvious (Figure 6A–B and Figure S7). After incubating the cells with PEG-CPP2@NPs, the strongest green fluorescence appeared in Lovo cells, and no significant green fluorescence was observed in HepG2, MCF-7, and HL-7702 cells (Figure 6C and Figure S7). This indicates that PEG-CPP2@NPs have high targeting ability to Lovo cells. In addition, the uptake of CCM, CCM@ZIF-90, and PEG-CPP2@NPs by Lovo cells was quantitatively measured using flow cytometry. As shown in Figure 6D and Fig. S8, PEG-CPP2-modified drug-loaded nanoparticles exhibited the strongest fluorescence intensity in Lovo cells and were more efficiently taken up by tumor cells than CCM and CCM@ZIF-90, while cells have the least uptake of free drug CCM. The results indicated that the loading of ZIF-90 and the modification of PEG-CPP2 favored the cellular uptake of the drug. CPP2 in particular further promotes the internalization of NPs in Lovo cells through the intracellular pathway of dynamin-dependent and reticular protein independent (Wang et al. 2023).

**Figure 6.**
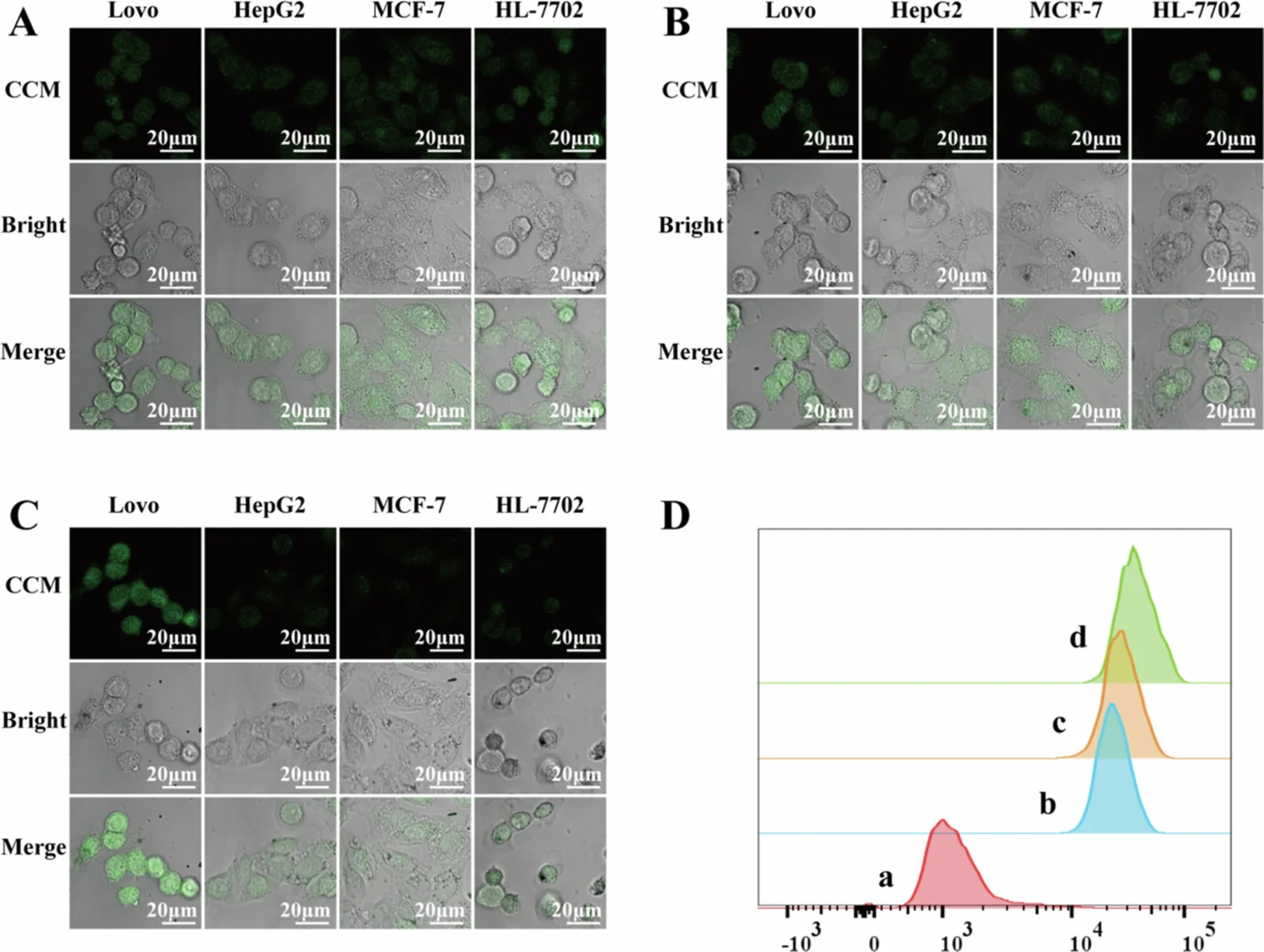
CLSM images of (A) CCM, (B) NPs, and (C) PEG-CPP2@NPs, (D) Flow cytometry quantitatively analyzed the Lovo cells uptake of (a) Control, (b) CCM, (c) NPs, and (d) PEG-CPP2@NPs.

### 3.6. *In vitro* cell efficacy evaluation

The good biocompatibility of nanomaterials is the prerequisite for their role in drug delivery. The safety of ZIF-90 was evaluated by MTT, Annexin V-FITC/PI staining, and DAPI staining. As shown in the result, within the scope of experimental concentration (0–80 μg/mL), different doses of ZIF-90 had a weak influence on Lovo cell viability (Figure 7A), and ZIF-90 did not induce cell apoptosis (Figure S5). The cell nuclei of each dose group were round with clear edges, and there was no significant difference compared with those of the control group (Figure S6). These results indicate that ZIF-90 has a good biosafety profile within the range of administration.

Subsequently, the cytotoxicity of the NPs to Lovo cells was evaluated using MTT assay and apoptosis assay. Figure 7B and C show that the inhibition of cell viability in all the drug preparation groups (CCM, NPs, and PEG-CPP2@NPs) was dose-dependent, and the drug preparation groups showed stronger cytotoxicity after laser irradiation, indicating that CCM chemotherapy and PDT synergistic therapy has a stronger anti-tumor effect. Regardless of laser irradiation, PEG-CPP2@NPs had a stronger inhibitory effect on tumor cell proliferation than CCM and NPs. This may be because cell-penetrating peptides have strong transmembrane transport ability, which helps their modified drug-loading NPs to enter and kill tumor cells (Ramsey and Flynn 2015). The effects of different preparations on apoptosis were detected by flow cytometry. As shown in Figure 7D, in the absence of laser irradiation, the apoptosis rates of free CCM, NPs, and PEG-CPP2@NPs were 40.23%, 48.96%, and 67.13%, respectively. After laser irradiation, the apoptosis rate of all the drug preparation groups was significantly higher than that of non-laser irradiation group, showing obvious photodynamic effect. The apoptosis rate of PEG-CPP2@NPs was 90.70%, and the promotion of apoptosis was the most significant.

PDT mainly induces the production of ROS to kill cells (Correia et al. 2021), and DCFH-DA can react with intracellular ROS to produce green fluorescence. The effect of laser irradiation on ROS production in various drug preparation groups was detected by DCFH-DA fluorescent probe. As shown in Figure 7E and F, in the absence of laser irradiation, all drug preparation groups promoted the production of ROS in tumor cells. Among them, PEG-CPP2@NPs induced the most ROS, which was 1.78 times that induced by CCM. After laser irradiation, more obvious green fluorescence was observed in Lovo cells incubated with each drug preparation group, and the intracellular ROS level was significantly higher than that without laser irradiation. The ROS levels in CCM, NPs, and PEG-CPP2@NPs increased by 68%, 83%, and 94%, respectively, after irradiation, indicating that PDT induces tumor cells to produce more ROS. A large number of intracellular ROS usually induces mitochondrial damage, leading to changes in the mitochondrial membrane potential (MMP), which promotes tumor cell apoptosis (Liu et al. 2023). The JC-1 fluorescent probe can detect the changes in MMP by red and green fluorescence. The MMP of normal cells is generally higher, and JC-1 can accumulate in mitochondria to produce red fluorescence. When cells undergo apoptosis, the MMP decreases, and JC-1 cannot accumulate, mainly in the form of green fluorescent monomer (Elefantova et al. 2018). The red–green fluorescence ratio of JC-1 in Lovo cells after incubation with each drug preparation was measured by flow cytometry. Figure 7G showed that the red‒green fluorescence ratio of JC-1 in each drug preparation group was lower than that in the control group, suggesting that CCM and its nanoformulations may induce mitochondrial damage in cells. After laser irradiation, MMP in each group was significantly lower than that without laser irradiation, which may be due to the fact that laser irradiation promoted the production of ROS and thus increased the damage to mitochondria.

In addition, flow cytometry was used to detect the effects of blank materials and drug preparations on the cell cycle. The results, as shown in Figure 7H and Figure S9, show that there was no difference in the cell cycle between Lovo cells treated with blank material ZIF-90 and the control group. After Lovo cells interacted with CCM and NPs, the cell cycle appeared G2/M phase arrest. After interaction with PEG-CPP2@NPs, cell cycle arrest appeared in the S phase, indicating that all drug preparations can induce the change of Lovo cell cycle and thus affect cell proliferation.

**Figure 7.**
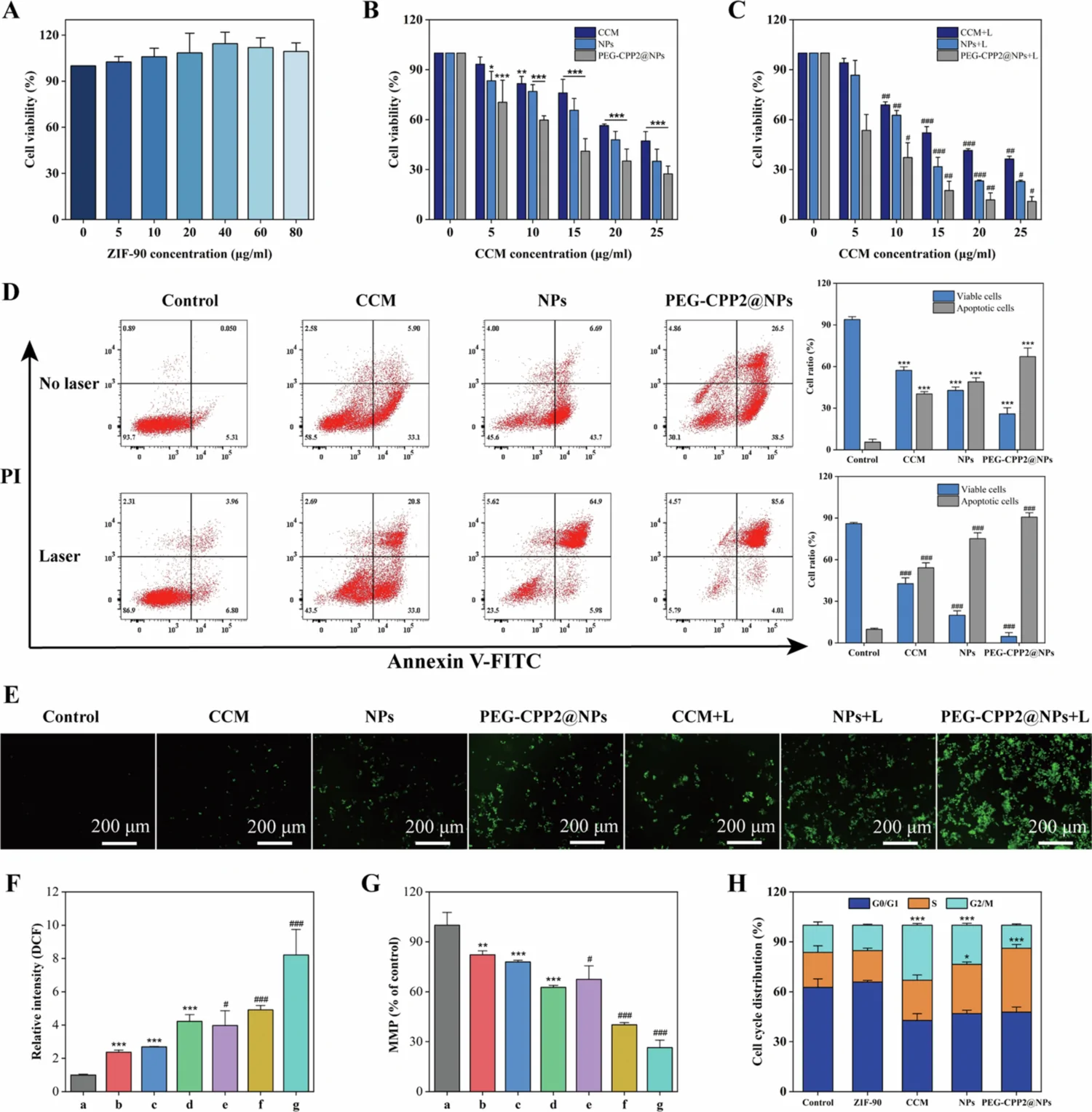
*In vitro* anti-tumor studies of nanoparticles. (A) Lovo cells viability after treatment with ZIF-90, (B) Lovo cells viability after various treatments without laser irradiation, (C) Lovo cells viability after various treatments with laser irradiation, (D) Apoptosis of Lovo cells was induced after various treatments in the absence or presence of laser irradiation, (E) Fluorescence microscopy imaging of ROS induced after various treatments with or without laser irradiation, (F) Quantitative analysis of ROS production after various treatments using flow cytometry, (a) saline, (b) CCM, (c) NPs, (d) PEG-CPP2@NPs, (e) CCM + L, (f) NPs + L, (g) PEG-CPP2@NPs + L, (G) Changes in MMP after various treatments were detected by flow cytometry with or without laser irradiation, (H) The effects of various treatments on Lovo cells cycle were analyzed by flow cytometry (*n* = 3, *P < 0.05, **P < 0.01, ***P < 0.001, compared to control, ^#^ *P* < 0.05, ^##^ *P* < 0.01, ^###^ *P* < 0.001, compared to the respective non-laser irradiation group).

### 3.7. *In vivo* biodistribution study

The biological distribution and tissue selection of nanoparticle preparations in mice carrying Lovo tumors were evaluated using a small animal live fluorescence imaging system. The biodistribution of NPs in the mice was observed at 0, 2, 4, 6, 12, and 24  h after tail intravenous injection of Cy5.5-CCM@ZIF-90 and PEG-CPP2/Cy5.5-CCM@ZIF-90, and the tumors and major organs of the mice were collected at 24  h after injection for *ex vivo* fluorescence imaging analysis. As shown in Figure 8A, PEG-CPP2/Cy5.5-CCM@ZIF-90 showed strong fluorescence at the tumor site of mice after 6 h of injection, whereas the fluorescence of Cy5.5-CCM@ZIF-90 was mainly distributed in the abdomen of mice. At 6 h post-injection, the fluorescence value of PEG-CPP2/Cy5.5-CCM@ZIF-90 in the tumor was the largest (Figure 8B), which indicated that the optimal time point for laser irradiation to evaluate the anti-tumor efficacy in vivo was 6 h of tail vein injection.

In addition, the results of *ex vivo* fluorescence imaging and quantitative analysis showed (Figure 8A and C) that the accumulation of PEG-CPP2-modified nanoparticles at tumor tissues was significantly higher than that at Cy5.5-CCM@ZIF-90, while PEG-CPP2/Cy5.5-CCM@ZIF-90 decreased distribution within the kidney and liver compared with Cy5.5-CCM@ZIF-90. This suggests that surface modification with PEG-CPP2 facilitates increased accumulation of NPs in tumor tissues, while simultaneously improving biocompatibility and mitigating uptake by the reticuloendothelial system. However, it should be noted that *in vivo* optical biodistribution alone cannot definitively distinguish CPP2-mediated active cell-specific targeting from passive accumulation governed by the enhanced permeability and retention (EPR) effect and PEG-promoted prolonged blood circulation.

**Figure 8.**
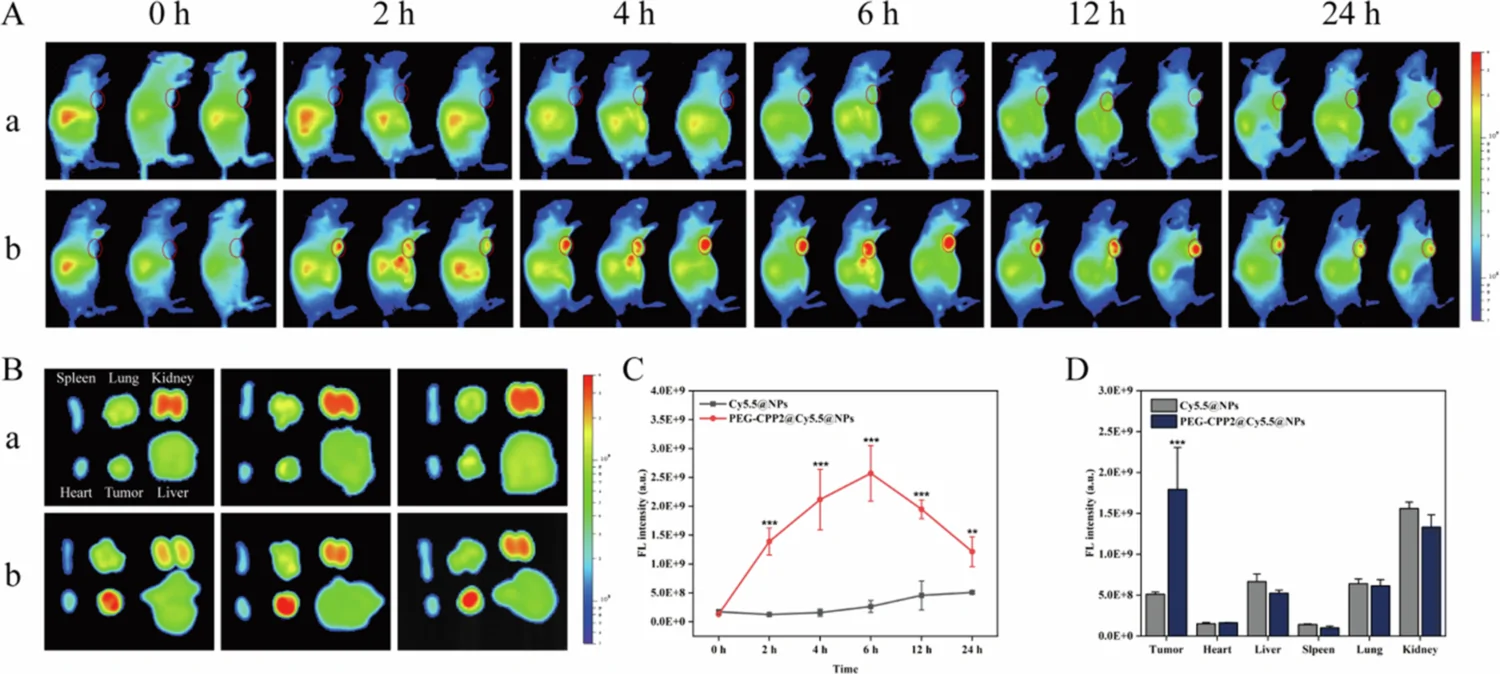
*In vivo* biodistribution of nanoformulations. (A) *In vivo* fluorescence imaging at different time points after intravenous NPs injection, (B) Fluorescence imaging of major organs and tumors at 24 h after injection, (C) Fluorescence intensity statistics in mice at different time points, (D) Quantitative analysis of fluorescence intensity in major organs and tumors of mice after 24 h injection, (a) Cy5.5@NPs, (b) PEG-CPP2@Cy5.5@NPs (*n* = 3, *P < 0.05, **P < 0.01, ***P < 0.001).

### 3.8. *In vivo* anti-tumor effect

Lovo tumor-bearing nude mice models were established to further investigate the in vivo anti-tumor effect of PEG-CPP2 modified NPs. The tumor-bearing mice were randomly divided into 7 groups: the control group was injected with saline intravenously; the non-light groups were injected with CCM, NPs, and PEG-CPP2@NPs (CCM dose: 7.5  mg/kg); and the tumors in the light groups were irradiated at 6 h after administration. Tail intravenous injection and laser irradiation were given on alternate days, and the volume of the tumors was recorded for 8 consecutive days. The changes of tumor volume in each group are shown in Figure 9A‒C. The tumor inhibition rates in each treatment were 25.46% (CCM), 33.16% (NPs), 43.5% (CCM+L), 48.45% (NPs+L), 66.05% (PEG-CPP2@NPs), and 80.64% (PEG-CPP2@NPs+L), respectively. All treatment groups had an anti-tumor effect compared with the control group, and the inhibition effects on tumor growth with laser irradiation groups were stronger than those of the non-irradiation groups, indicating that chemotherapy and PDT synergistic treatment had stronger anti-tumor efficacy. PEG-CPP2@NPs revealed a more significant tumor inhibition effect than free CCM and NPs, which may be due to the loading of ZIF-90 reducing the clearance rate of free CCM *in vivo*, and the modification of PEG-CPP2 promoting the specific targeting of drug-loading NPs on Lovo cells. This increased the accumulation of drug-carrying NPs in tumor tissue, and thus, the PEG-CPP2@NPs laser irradiation treatment group showed the strongest tumor suppression potency.

In addition, HE and TUNEL staining were performed for pathological analysis of tumor tissues, and TUNEL staining was quantitatively analyzed (Figure 9D‒F). Compared with other groups, PEG-CPP2@NPs+L group possessed the most serious cell nucleus shrinkage, disruption, or disappearance, and the strongest TUNEL fluorescence, suggesting the tumor tissue necrosis was obvious, and the cell apoptosis was very densest, which had superior anti-tumor effects. As a photosensitizer of natural sources, CCM has obvious photodynamic properties (Ailioaie et al. 2021). A DHE fluorescent probe was used to detect ROS in tumor tissues (Figure 9G). Both CCM and its nanomaterials can induce ROS generation, and ROS production in tumor tissues increased significantly after laser irradiation, indicating that ROS levels *in vivo* improved during PDT.

The anti-tumor mechanism of CCM may be mainly related to inducing apoptosis of tumor cells and inhibiting cell proliferation, reportedly, which can be achieved through the regulation of multiple pathways, including Bax, Bcl-2, Cytochrome C, caspase-3, and so on (Ismail et al. 2019; Wang et al. 2023). The up-regulated expression of Bax and down-regulated expression of Bcl-2 can change the permeability of the mitochondrial membrane, thus promoting the release of Cytochrome C. A large amount of Cytochrome C is able to induce a cascade reaction of Caspase pathway, such as activation of apoptosis-executive protein Caspase-3, leading to apoptosis of tumor cells. The levels of Bax, Bcl-2, Cytochrome C, and caspase-3 in mice tumor tissues were detected by Western blotting to explore the possible mechanism of CCM in promoting tumor cell apoptosis. As shown in Figure 9H-K, the Bax/Bcl-2 ratio, Cytochrome C, and caspase-3 expressions in all drug preparation groups with laser irradiation were obviously enhanced compared to those in non-irradiation groups, and the protein levels in the PEG-CPP2@NPs group were significantly higher than those in the free CCM and NPs groups.

The above data support that the drug-loaded NPs functionalized with PEG-CPP2 can accumulate within tumor tissues and exert pronounced anti-tumor effects via combined chemo-photodynamic actions. The observed up-regulation of the Bax/Bcl-2 ratio, Cytochrome C release, and caspase-3 activation, alongside elevated ROS production and mitochondrial disruption, indicates the activation of the mitochondrial apoptotic cascade. Nevertheless, because pathway-specific pharmacological inhibitors or molecular blocking experiments were not implemented in the current study, these observations should be viewed as correlative evidence supporting pathway involvement. In addition, two translational constraints warrant explicit consideration. First, as noted, the animal cohort size of *n* = 4 per treatment group constitutes an inherent limitation regarding statistical robustness for broad preclinical extrapolation. Second, the therapeutic excitation used herein relies on a 450-nm blue laser. While 450 nm aligns well with the characteristic absorption profile of curcumin, its tissue penetration depth is inherently restricted compared to near-infrared (NIR) light, primarily limiting its direct clinical utility to superficial lesions. For the translation of this platform to deeper colorectal tumors, the integration of fiber-optic colonoscopic delivery systems or future adaptations involving red/NIR-shifted photosensitizing conjugates will be essential.

**Figure 9.**
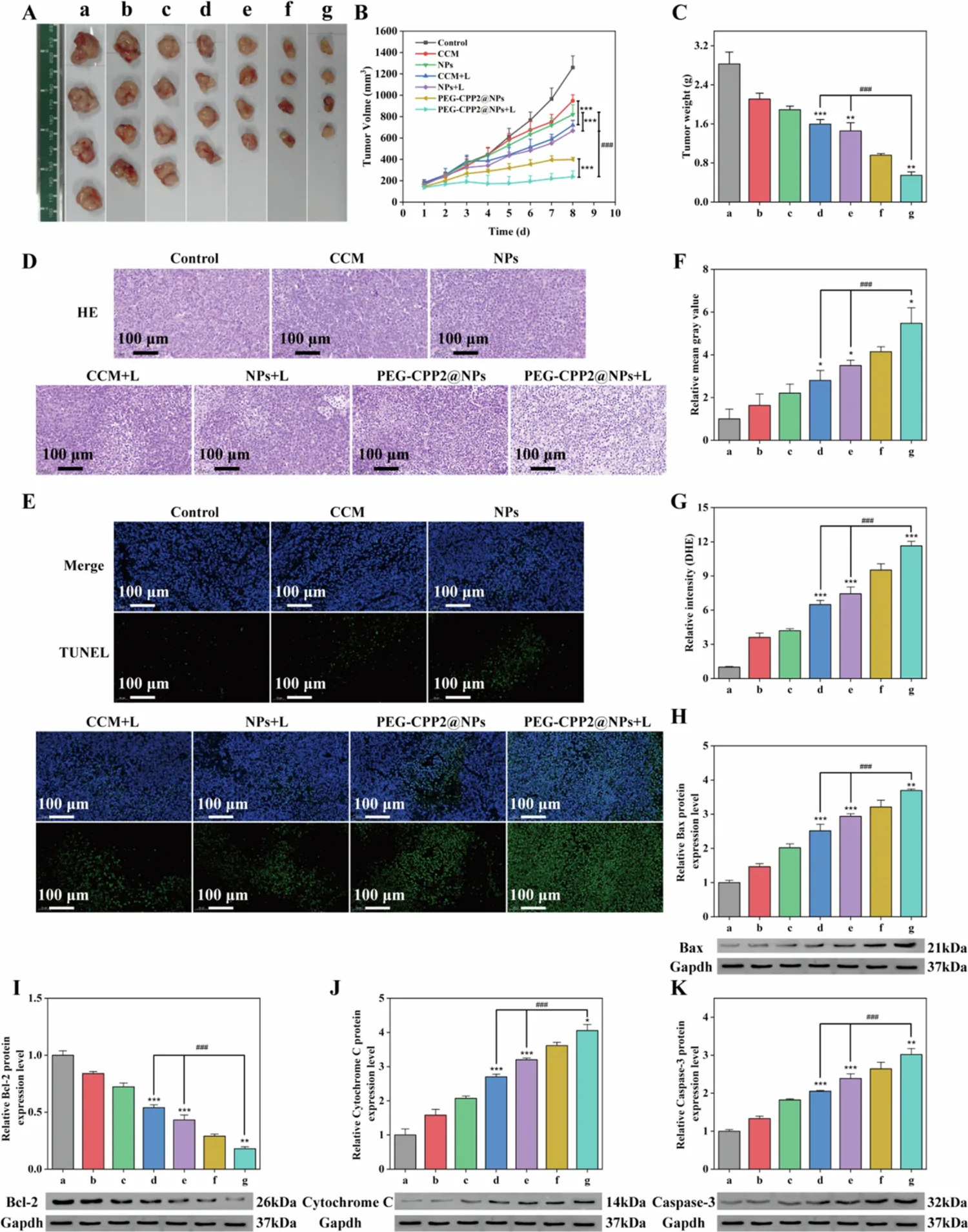
*In vivo* anti-tumor effect of nanoparticles. (A) Photographs of Lovo tumors collected after various treatments, (a) saline, (b) CCM, (c) NPs, (d) PEG-CPP2@NPs, (e) CCM+L, (f) NPs+L, (g) PEG-CPP2@NPs+L (*n* = 4), (B) tumor volume changes in mice after various treatments without or with laser irradiation (*n* = 4), (C) the weight of tumor tissues after the end of treatments (*n* = 4), HE staining (D) and TUNEL staining (E) of tumor tissues after various treatments (scale bars: 100 μm), (F) Quantitative analysis of TUNEL staining, (G) ROS production detection in tumor tissues, The expression of Bax (H), Bcl-2 (I), Cytochrome C (J) and caspase-3 (K) in tumor tissues (*n* = 3, *P < 0.05, **P < 0.01, ***P < 0.001, compared to the respective non-laser irradiation group; ^#^ *P* < 0.05, ^##^ *P* < 0.01, ^###^ *P* < 0.001, compared to PEG-CPP2@NPs+L group).

### 3.9. *In vivo* biosafety evaluation

*In vivo* biosafety is an important prerequisite for the efficacy of nanomedicines (Santos et al. 2014). The biological safety of each group was evaluated by observing the changes of body weight during treatment, serum biochemical parameters and HE staining sections of major organs after treatment. Figure 10A shows that the weight of the mice did not change significantly during drug administration. The liver and kidney are the main organs for drug metabolism and excretion, and nano-formulations inevitably accumulate in the liver and kidney (Kermanizadeh et al. 2014; Hauser et al. 2021). After treatment, the blood of the mice was collected for biochemical analysis. Compared with the control group, there were no obvious differences in ALT, AST, ALP, BUN, and CRE levels among all groups, which indicated that the drug preparations did not cause significant damage to liver and kidney function (Figure 10B). Besides, the major organs of the heart, liver, spleen, lung, and kidney were collected for HE staining after the mice were sacrificed. As shown in Figure 10C, no evident histopathological differences were observed in the main organs of treatment groups compared with the control group, suggesting that CCM and its nano-preparations had no distinct toxicity to the major organs of mice. The above data confirmed that nano-preparations had good biosafety *in vivo* and possessed potential for the treatment of tumors.

**Figure 10.**
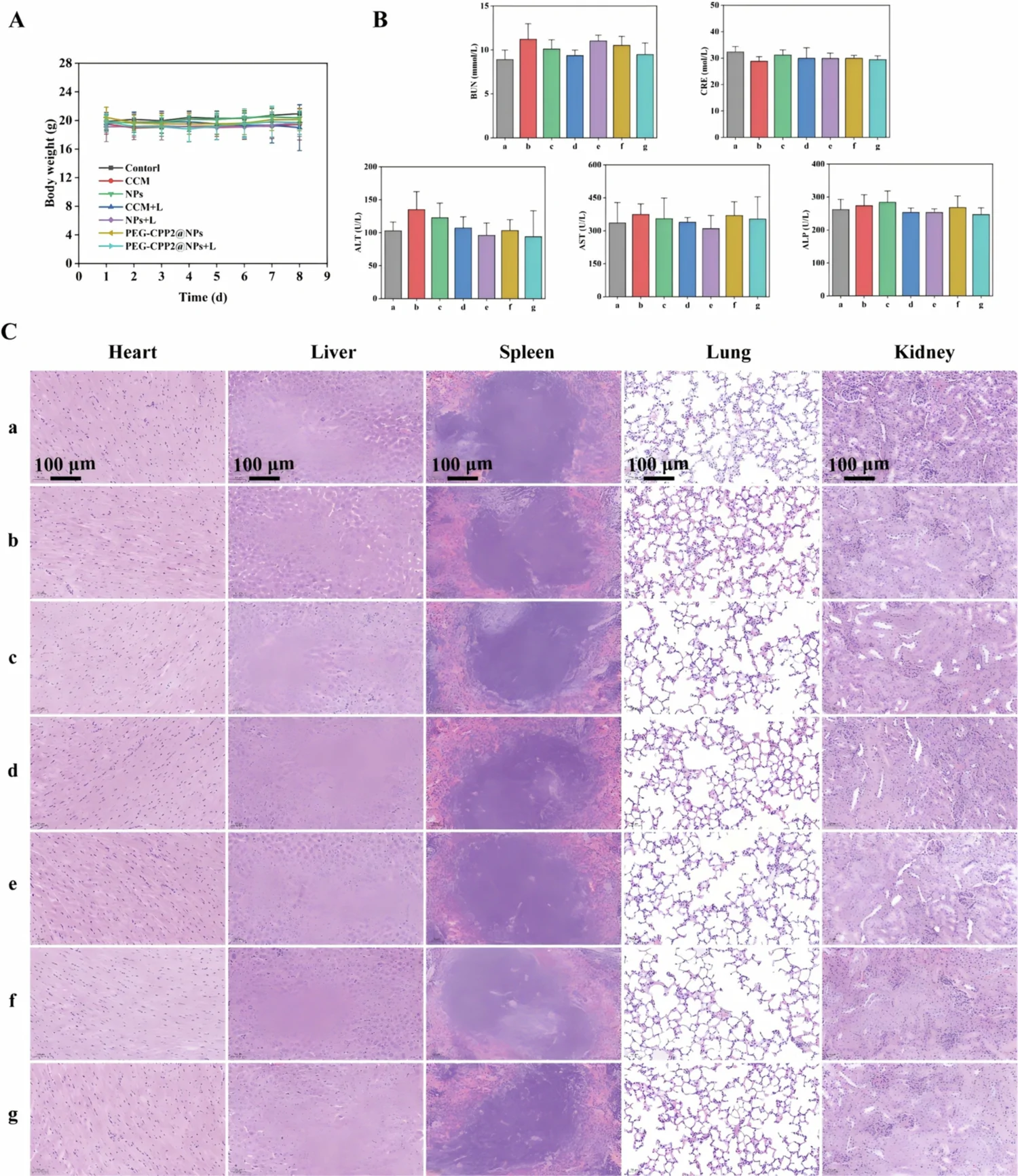
*In vivo* biosafety evaluation of nanoparticles. (A) Body weight changes in mice after various treatments without or with laser irradiation (*n* = 4), (B) BUN, CRE, ALT, AST and ALP levels in serum of mice after various treatments (*n* = 3), (C) The HE images of heart, liver, spleen, lung and kidney of mice at the end of various treatments (*n* = 3), (a) saline, (b) CCM, (c) NPs, (d) PEG-CPP2@NPs, (e) CCM+L, (f) NPs+L, (g) PEG-CPP2@NPs+L.

## 4. Conclusion

We constructed a targeted drug delivery nanoplatform to exploit the combined anti-tumor effects of curcumin in chemotherapy and PDT. CCM was loaded into the caged cavity of ZIF-90 to improve the stability of free CCM, and PEGylated CPP2 was modified to the surface of the drug-loading particle by the reaction coating method. The prepared multifunctional nanoparticle had good drug loading ability and excellent photodynamic properties *in vitro*, and produced abundant ROS after laser irradiation. The surface modification with the targeting ligand PEG-CPP2 promoted the uptake of drug-loading nanoparticles by Lovo tumor cells, which was conducive to the chemotherapy effect of CCM and significantly advanced the apoptosis of tumor cells. The *in vitro* and *in vivo* results showed that the nanoparticles substantially elevated ROS generation, exacerbated mitochondrial dysfunction, and promoted apoptotic signaling following laser irradiation, suggesting that the integration of CCM chemotherapy and PDT produces a potent cooperative anti-tumor response. Experimentally, this engineered ZIF-90 platform solves key translational bottlenecks of free CCM by achieving exceptionally high loading, pH-responsive release, enhanced photostability, and preferential uptake in colorectal tumor cells, culminating in marked tumor growth suppression *in vivo*. Nonetheless, definitive verification of the exact causal apoptotic pathways via molecular inhibition, validation across larger animal cohorts, and circumvention of the tissue penetration limits of 450-nm light (e.g. through endoscope-guided illumination) will be valuable directions for future clinical translation. In addition, the functional nanoparticles possessed good biocompatibility and were able to increase the killing effect on tumor cells without significant damage to normal tissues. Overall, this study designed targeted drug delivery nanoparticles based on CCM chemotherapy combined with PDT treatment, providing a reference scheme for improving the anti-tumor efficacy of CCM and targeted delivery of anti-colon cancer drugs. However, this study did not explore the immunomodulatory potential of the CCM combination therapy platform. For subsequent research, we consider using immunoactive or humanized mouse models to verify whether this therapy can increase the infiltration of cytotoxic T cells in tumors, alter the immunosuppressive cell population, and explore its synergistic potential when used in combination with immunotherapies such as immune checkpoint inhibitors.

## Supplementary Material

FigureS1.tif FigureS1.tif

FigureS2.tif FigureS2.tif

FigureS3.tif FigureS3.tif

FigureS4.tif FigureS4.tif

FigureS5.tif FigureS5.tif

FigureS6.tif FigureS6.tif

FigureS7.tif FigureS7.tif

FigureS8.tif FigureS8.tif

FigureS9.tif FigureS9.tif

Supporting Information.docx Supporting Information.docx

Author Checklist.pdf Author Checklist.pdf

## Data Availability

The datasets used and/or analyzed during the current study are available from the corresponding author on reasonable request.
