# Supplementary material for: Cell-penetrating peptide functionalized ZIF-90 platform for photodynamic-chemical combined therapy in colorectal cancer
Source: Drug Deliv. 2026 Nov 7;33(1):2746812. doi: 10.1080/10717544.2026.2746812 (PMC13757870; doi:10.1080/10717544.2026.2746812)
Supplement: Supplementary file 1 [file IDRD_A_2746812_SM0358.pdf]

NOTE: Please save this file locally before filling in the table, DO NOT work on the file within your internet browser as changes will not be saved. Adobe Acrobat Reader (available free here) is recommended for completion.

The ARRIVE guidelines 2.0: author checklist

The ARRIVE Essential 10

These items are the basic minimum to include in a manuscript. Without this information, readers and reviewers cannot assess the reliability of the findings.

Item		Recommendation	Section/line number, or reason for not reporting
**Study design**	1	For each experiment, provide brief details of study design including:	
		a. The groups being compared, including control groups. If no control group has been used, the rationale should be stated.	Line 267, 270 and 271
		b. The experimental unit (e.g. a single animal, litter, or cage of animals).	A single animal
**Sample size**	2	a. Specify the exact number of experimental units allocated to each group, and the total number in each experiment. Also indicate the total number of animals used.	Line 265 to 273
		b. Explain how the sample size was decided. Provide details of any *a priori* sample size calculation, if done.	Line 263
**Inclusion and exclusion criteria**	3	a. Describe any criteria used for including and excluding animals (or experimental units) during the experiment, and data points during the analysis. Specify if these criteria were established *a priori*. If no criteria were set, state this explicitly.	Line 264
		b. For each experimental group, report any animals, experimental units or data points not included in the analysis and explain why. If there were no exclusions, state so.	No exclusion
		c. For each analysis, report the exact value of *n* in each experimental group.	Line 266 to 272
**Randomisation**	4	a. State whether randomisation was used to allocate experimental units to control and treatment groups. If done, provide the method used to generate the randomisation sequence.	Line 290
		b. Describe the strategy used to minimise potential confounders such as the order of treatments and measurements, or animal/cage location. If confounders were not controlled, state this explicitly.	Strict group processing
**Blinding**	5	Describe who was aware of the group allocation at the different stages of the experiment (during the allocation, the conduct of the experiment, the outcome assessment, and the data analysis).	not applicable
**Outcome measures**	6	a. Clearly define all outcome measures assessed (e.g. cell death, molecular markers, or behavioural changes).	Line 624
		b. For hypothesis-testing studies, specify the primary outcome measure, i.e. the outcome measure that was used to determine the sample size.	Line 262
**Statistical methods**	7	a. Provide details of the statistical methods used for each analysis, including software used.	Line 307 to 309
		b. Describe any methods used to assess whether the data met the assumptions of the statistical approach, and what was done if the assumptions were not met.	Line 310
**Experimental animals**	8	a. Provide species-appropriate details of the animals used, including species, strain and substrain, sex, age or developmental stage, and, if relevant, weight.	Line 258 to 260
		b. Provide further relevant information on the provenance of animals, health/immune status, genetic modification status, genotype, and any previous procedures.	Line 260 to 263
**Experimental procedures**	9	For each experimental group, including controls, describe the procedures in enough detail to allow others to replicate them, including:	
		a. What was done, how it was done and what was used.	Section 2.15 and 2.16
		b. When and how often.	Section 2.15 and 2.16
		c. Where (including detail of any acclimatisation periods).	Section 2.14
		d. Why (provide rationale for procedures).	Line 268 and 272
**Results**	10	For each experiment conducted, including independent replications, report:	
		a. Summary/descriptive statistics for each experimental group, with a measure of variability where applicable (e.g. mean and SD, or median and range).	Section 3.8
		b. If applicable, the effect size with a confidence interval.	not applicable
