# Supplementary material for: Cell-penetrating peptide functionalized ZIF-90 platform for photodynamic-chemical combined therapy in colorectal cancer
Source: Drug Deliv. 2026 Nov 7;33(1):2746812. doi: 10.1080/10717544.2026.2746812 (PMC13757870; doi:10.1080/10717544.2026.2746812)
Supplement: Supplementary file 2 [file IDRD_A_2746812_SM9195.docx]

Supporting Information

### Cell-Penetrating Peptide Functionalized ZIF-90 Platform for Synergistic Photodynamic-chemical Combined Therapy in Colorectal Cancer by Amplifying ROS Generation

Tingting Fu^a,b,1^, Boran Ni^a,1^, Dongge Yin^b^, Rongyue Zhu^b^, Shilang Liao^b^, Yuji Du^b^, Xiaohong Jing^b^, Yufei Sun^b^, Xixi Deng^b^, Chenyao Wu^b^, Xiwen Cao^b^, Mengru Cai^c,^*, Xingbin Yin^b,^*

a Department of Endocrinology, Guang’ Anmen Hospital of China Academy of Chinese Medical Sciences, Beijing, China (100053)

b School of Chinese Materia Medica, Beijing University of Chinese Medicine, Fangshan District, Beijing, China (102488)

c Institute of Basic Theory for Chinese Medicine, China Academy of Chinese Medical Sciences, Beijing, 100700, China (100700)

Corresponding author:

E-mail addresses: cmr199711@163.com (M. Cai), yxbtcm@bucm.edu.cn (X. Yin)

^1^ These authors contributed equally to this work.

**Figure S1.** Particle size diagram of ZIF-90

**Figure S2.** Particle size diagram of CCM@ZIF-90

**Figure S3.** Particle size diagram of PEG-CPP2/CCM@ZIF-90

**Figure S4.** Zata potential of (A) CCM@ZIF-90 and (B) PEG-CPP2/CCM@ZIF-90

**Figure S5.** Cell apoptosis rates after treatment with various concentrations of ZIF-90. (A) Control; (B) 5 μg/mL; (C) 40 μg/mL; (D) 80 μg/mL.

**Figure S6.** DAPI staining fluorescence images of Lovo cells were treated with various concentrations of ZIF-90.

**Figure S7.** Quantitative analysis of CLSM images

**Figure S8.** Flow cytometry quantitatively analyzed the Lovo cells uptake of (a) Control (b) CCM (c) NPs and (d) PEG-CPP2@NPs.

**Figure S9.** Diagrams of Lovo cell cycle after different treatments

**Table S1.** Factors of orthogonal experiment

| Factor level | A  Drug loading Time(h) | B  Ratio (drug:carrier) | C  Concentration (mg/mL) |
| --- | --- | --- | --- |
| 1 | 12 | 1:1 | 2 |
| 2 | 24 | 2:1 | 3 |
| 3 | 48 | 4:1 | 4 |

**Table S2.** Drug loading orthogonal

|  | A  Drug loading Time(d) | B  Ratio (drug:carrier) | C  Concentration (mg/mL) | D  blank | Drug loading  (%) |
| --- | --- | --- | --- | --- | --- |
| F1 | 1 | 1 | 1 | 1 | 18.40 |
| F2 | 1 | 2 | 2 | 2 | 25.95 |
| F3 | 1 | 3 | 3 | 3 | 34.21 |
| F4 | 2 | 1 | 2 | 3 | 30.49 |
| F5 | 2 | 2 | 3 | 1 | 26.93 |
| F6 | 2 | 3 | 1 | 2 | 44.40 |
| F7 | 3 | 1 | 3 | 2 | 33.84 |
| F8 | 3 | 2 | 1 | 3 | 44.90 |
| F9 | 3 | 3 | 2 | 1 | 54.74 |
| K1 | 26.177 | 27.568 | 35.890 |  |  |
| K2 | 33.940 | 32.595 | 37.062 |  |  |
| K3 | 44.496 | 44.450 | 31.661 |  |  |
| R | 18.319 | 16.881 | 5.401 |  |  |

**Table S3.** Orthogonal test variance analysis

|  | Sum of Squares | df | F | P |
| --- | --- | --- | --- | --- |
| A | 507.276 | 2 | 33.084 | 0.029 |
| B | 450.790 | 2 | 29.400 | 0.033 |
| C | 48.438 | 2 | 3.159 | 0.24 |

**Table S4.** Three batches of verification experiments

|  | Drug loading (%) | Average value (%) | RSD (%) |
| --- | --- | --- | --- |
| F1 | 54.84 | 55.24 | 1.94 |
| F2 | 56.45 |  |  |
| F3 | 54.42 |  |  |

Drug loading =$\frac{M1-M2}{M1-M2+M3}$× 100%

M1: the total amount of drug; M2: the un-loaded amount of drug in the supernatant; M3: the amount of ZIF-90
